# Biowaste Valorization of Palm Tree *Phoenix dactylifera* L. for Nanocellulose Production

**DOI:** 10.1049/2024/7867463

**Published:** 2024-05-27

**Authors:** Randa Mohammed Dhahi, Mohammed Majeed Mohammed, Haitham Mawlood Mikhlif

**Affiliations:** ^1^ Department of Biology College of Education Al-Iraqia University, Baghdad, Iraq; ^2^ Department of Physics College of Science Mustansiriyah University, Baghdad, Iraq

## Abstract

The desire to reduce reliance on oil resources arises from the concerns about carbon footprint and nonrenewability. Conversely, the global presence of over 100 million palm trees poses a significant challenge due to the substantial amount of biowaste generated annually. Additionally, the use of nanocellulose (NC) as a cost-effective material is steadily gaining recognition for its growing adaptability over time. The main goal of this study is to biosynthesized NC from Iraqi date palm *Phoenix dactylifera* leaves waste with low-concentration acid-alkali treatment. The date palm leaves waste yields 20 g of NC from 100 g of leaves before acid hydrolysis treatment. The chemical components of biosynthesized NC were 47.90%, 26.78%, and 24.67% for *α*-cellulose, hemicellulose, and lignin, respectively. In order to study their properties, NC from raw date palm leaves was studied by microscopic techniques such as scanning electron microscopy (SEM), energy dispersive X-ray (EDX) spectroscopy, and atomic force microscope (AFM). SEM results revealed rod-like structured NC as well as combined long-fine fibrous structures rather than compacted bundles with sizes ranging between 31 and 74 nm. With EDX, all spectra exhibit the peaks of carbon and oxygen as the main elements with 63.8% and 10.44%, respectively, in their compositions, which relate to the typical composition of cellulose. The 3D image of AFM NC with a tapping mode presented a highly uniform distribution of NC with a size of ∼15 nm. The statistical roughness analysis shows that the obtained roughness average is 7.20 nm with the root–mean-square roughness value of 21.56 nm, which corresponded relatively with the micrographs of SEM. The results of this study demonstrate the promise of using date palm waste as raw material to produce NC as green nanocomposite from biodegradable nanomaterials for water purification and sustained drug delivery for biomedical applications. In this regard and because of the insufficient reports about the extraction of NC from palm tree leaves waste, the objective of this study was designed to fabricate NC biologically from fibers sourced from the waste of Iraqi date palm *P. dactylifera* leaves that left in agricultural lands or burned, which can be an ecological and health problem as a bionanocomposites in the medical and industrial field and as alternative resources of wood materials.

## 1. Introduction

The increased focus on the utilization of natural biopolymer fibers, as opposed to synthetic fibers, has prompted researchers to investigate various sources of biodegradable natural fibers. Among the available options, cellulose stands out as the most abundant renewable biopolymer fiber. Cellulose is primarily composed of D-glucose and consists of a linear and unbranched homopolysaccharide structure. It is worth noting that this biopolymer exhibits remarkable thermal and mechanical stabilities, while also demonstrating minimal to no toxicity toward the environment [1].

With an estimated annual production of ∼7.5 × 10^10^ tons, cellulose can be considered potentially the most abundant natural biopolymer found on our planet. Cellulose, a high-molecular-weight homopolymer, is comprised of repeating units of *β*-d-glucopyranosyl that are connected through (1–4) glycoside linkages and can be extracted from various biomasses [2]. Cellulose is composed of both crystalline and amorphous regions. By employing acid hydrolysis treatment [3], the amorphous area of cellulose can be removed, resulting in the formation of crystalline particles known as nanocellulose (NC) or cellulose nanocrystals (CNCs) that exhibit noteworthy attributes such as cost-effectiveness, non-toxicity, excellent thermal stability, optical transparency, and biodegradability [4].

Due to its exceptional thermal and mechanical properties, NC is extensively employed as a reinforcing agent in polymer composites. The incorporation of NC significantly enhances the physicochemical, thermal, and insulating characteristics of various biodegradable polymers, thereby enabling their suitability for a wide range of applications [5]. Iraq, known as the originating hub of the date palm, holds historical significance as the site of domestication for this crop. Additionally, Iraq held the distinction of being the global leader in date production for a significant span of time. Both historical records and genetic testing provide evidence that Iraq serves as the birthplace of the date palm, scientifically known as *Phoenix dactylifera*. This country, since 4,000 B.C., has witnessed the domestication and cultivation of this tree. Moreover, it holds a significant historical background and the consumption of palm trees has always played a crucial role in its culture. These dates not only constitute a major agricultural product but also symbolize the identity of the nation, as they are depicted on our currency. The inhabitants of Iraq have extensively utilized this tree for sustenance, medicinal purposes, furniture manufacture, and an assortment of household utensils [6].

Each year, trees can produce about 200,000 tons of lignocellulosic waste after planting or fruit harvesting (branches, stalks, and trunks). Meanwhile, these agro-wastes can be used in the production of environmentally friendly materials with low costs for many applications, such as in textiles, baggage, sports items, and automotive parts [7, 8, 9]. In this regard, and because of the insufficient reports about the extraction of NC from palm tree leaves waste, the objective of this study was designed to fabricate NC biologically from fibers sourced from the waste of Iraqi date palm *P. dactylifera* leaves that left in agricultural lands or burned, which can be an ecological and health problem as a bionanocomposites in the medical and industrial field and as alternative resources of wood materials.

## 2. Material and Experimental

### 2.1. Materials and Chemicals

The leaves of Iraqi date palm *P. dactylifera* L. were collected from the north of Baghdad's Orchard. Sodium chlorite (80%) (Sigma-Aldrich), Sodium hydroxide (97%) (Sigma-Aldrich), acetic acid glacial (99.9%) (LTD), and sulfuric acid (95%–97%) (Sigma-Aldrich), all the materials were employed in their original form as acquired from the supplier.

### 2.2. Nanocrystalline Cellulose Extraction Process

#### 2.2.1. Cleansing Technique

**Figure 1:**
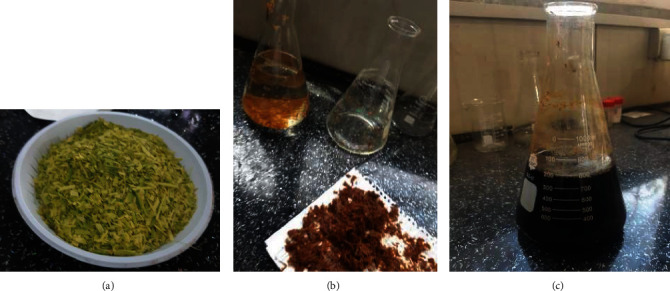
Nanocrystalline cellulose extraction process from date palm leaves waste: (a) cleansing; (b) alkaline treatment; (c) acid treatment.

Upon washing and cutting, the date palm fibers were mechanically crushed (Figure 1(a)) and subsequently submerged in water at a temperature of 100°C, while being stirred by a magnetic stirrer at 300 revolutions per minute for 1 hr, in order to eliminate any solutes present. Subsequently, these fibers were dried under vacuum conditions at a temperature of 50°C for a period of 15 hr.

#### 2.2.2. Alkaline Treatment

In order to obtain pure cellulose, the date palm fibers were subjected to a 4 wt.% sodium hydroxide (NaOH) solution for 2 hr at a temperature of 80°C. The resultant mixture was then filtrated and washed multiple times with distilled water, following which it was dried under vacuum conditions at a temperature of 50°C for a duration of 24 hr (Figure 1(b)). This treatment was repeated twice.

#### 2.2.3. Bleaching Treatment

Post-alkaline treatment, a 1.7 wt.% solution of sodium chlorite (NaClO_2_) was prepared, and acetic acid was added until the solution reached a pH of 4. Subsequently, the palm fibers were treated with this solution at a temperature of 80°C for a duration of 2 hr. The mixture was then filtrated using distilled water to procure white-colored fibers, which were subsequently dried in a vacuum oven at a temperature of 50°C for a duration of 24 hr. The treatment was achieved twice to purify the cellulose component, Figure 1(c).

#### 2.2.4. Acid Hydrolysis Treatment

The last step in nanocrystalline cellulose extraction was acid hydrolysis treatment. The pulp fiber was treated via 64 wt.% of sulfuric acid (H_2_SO_4_) solution at 45°C for 30 min under steady stirring. The resulted mixture was filtered and washed thoroughly with distilled water to ensure the removal of acid, and then it was dehydrated at 50°C in a vacuum oven for 24 hr (Figure 1(c)) [10].

### 2.3. Yield Determination

The NC yield was calculated using Equation (1), as given below:

(1) $$\begin{array}{c} \text{Yield }\left(\%\right)=\left(M1/M2\right)\times 100, \end{array}$$

in which M1 is the weight of oven-dried NC, and M2 is the weight of raw date palm.

### 2.4. The Chemical Compositions of NC

The chemical compositions of NC were determined, such as *α*-cellulose, hemicellulose, lignin, and according to Poddar et al. [11].

### 2.5. Microscopic Analysis

#### 2.5.1. Light Microscope

After acid hydrolysis treatment, the palm fibers with the biosynthesized nanofibrils were examined under a light microscope at 40x magnification after staining with eosin dye to confirm the decomposition of the fibers for NC production, which was confirmed using the scanning electron microscope (SEM).

#### 2.5.2. SEM Analysis

The morphology of chemically treated biomass was investigated using SEM (Hitachi) equipped energy dispersive X-ray (EDX); EDX was used to examine the composition of the elements that identified samples at 10,000 and 200,000× magnification. Dry powder sample was placed on carbon tapes and then sputtered with a thin gold layer under an argon atmosphere with an accelerating voltage at 6 kV, while elemental analysis by EDX was achieved at an accelerating voltage of 20 kV.

#### 2.5.3. Atomic Force Microscopy (AFM)

AFM (Nanosurf, Switzerland, V3.1R0, BT03710-15.) examination was used by dropping aqueous cellulosic nanoparticles steadily on a glass slide, and then it was dried by air before investigation. Then, images were captured by applying tapping mode on the NC samples with a scan rate of 4.80–6.00 *μ*m/s at ambient conditions.

## 3. Results

The Iraqi date palm biowaste yielded 20 g of cellulose from 100 g of oven-dried leaves before acid hydrolysis treatment; the yield percentage exhibited by this work is found to be comparable to the study by Mehanny et al. [12], showing the yield of fronds was 2.9 g. The percent of the chemical components of biosynthesized NC are shown in Table 1. The *α*-cellulose content of date palm was 47.9%, while hemicellulose was 26.78% from the total component, and the lignin was 24.67%. This result was possibly similar with the previous finding [13]. These dietary fibers have several features, such as natural, cost-effective, and as a natural source of biodegradable cellulose.

Cellulose is the most abundant polysaccharide in our planet because it is the main building compound for most plants. It is a linear homopolymer made up of glucan chains with repeating (1–4)-D-glucopyranose units connected by O-glycosidic bonds [13]. Various reports have been conducted on fibers derived from date palm trees as reinforcing materials; these efforts are ongoing to detect new resources of cellulose biomass as a feedstock instead of costly traditional industry, which is now limited in relation to conserving environments [14, 15, 16, 17, 18]. However, because of the COVID-19 lockdown, these byproducts became attracted publicly as alternative feedstuffs [19].

**Figure 2:**
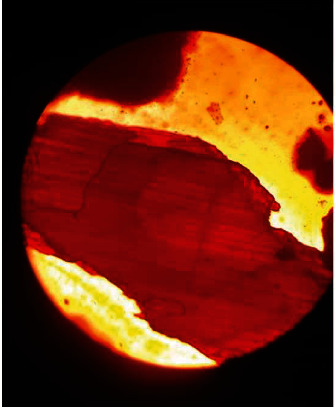
Illustrated the raw date palm leaves after acidic hydrolysis under light microscope with 40x magnification.

**Figure 3:**
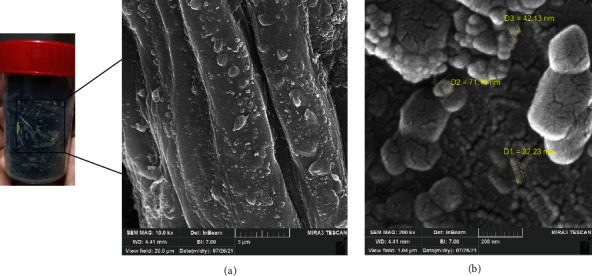
SEM images for the obtained nanocellulose with low (a) and high (b) magnification showing the shape and size of the obtained particles after the acidic hydrolysis process.

The size and morphology of NC extracted from the palm waste were demonstrated by the light compound and SEM microscopic analysis, as illustrated in Figures 2 and 3, respectively.  Figure 2 indicates the destruction of the plant cells, especially the fibers of the palm leaves, after acidic treatment and the production of NC. It is observed that palm leaf fibers were broken and appeared as irregular bundles, which indicates the possibility of producing cellulose at the nanoscale, confirmed by SEM analysis.

Chemical treatments are the public approaches that followed to solubilizing the molecules except cellulose [20]. Acid hydrolysis is the most known chemical protocol for NC extraction, the acid's protons diffuse into the fibers and specially react with the disarranged cellulose fibrils sections, which are more reachable, to split the glycosidic bonds. The crystalline regions were more resistant to degradation and this process takes a longer time, allowing the NC isolation wholly of crystalline sections. The acid breaks cellulosic chains at the glycosidic link, steadily lowering the cellulose polymerization degree until NC is succeeded [21].

Figures 3(a) and 3(b) illustrated the shape and distribution of NC, which were observed using SEM with different magnifications. As that SEM investigation is achieved on dry powder sample, the leaves residues NC show the swelling of cellulose was observed followed by isolation of rod-like structured cellulosic nanoparticles appeared as well as combined long-fine fibrous structures rather than a compacted bundle (Figure 3(a)), additionally to the high-intensity electron beam during SEM study splitting, with the acidic treatment, the nanoparticles had further broken into shorter crystallites with sizes ranging between 31 and 74 nm (Figure 3(b)). The presence of nanoscale cellulose suggests that the biosynthetic process was used successfully for date palm leaves waste with nanosized dimensions. These results are much better compared to some literature by Saud et al. [22].

The small particle size of biosynthesized NC was a promising starting material for industrial application. The width of NC for the current work was better in comparison to widths yielded by Abu-Thabit et al. [14] with 42–82 nm spherical particles.

**Figure 4:**
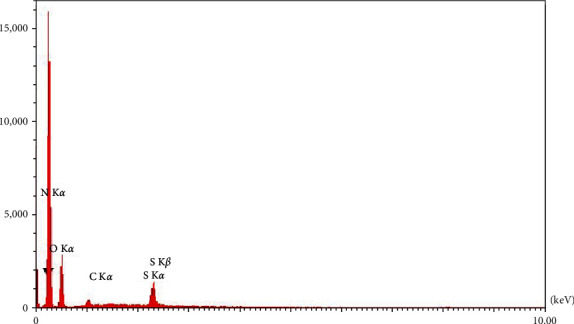
The EDX patterns of cellulosic nanoparticle biosynthesized from palme tree fronds.

The EDX analysis of palm tree fronds is shown in Figure 4 and Table 2. All EDX spectra exhibit the peaks of carbon and oxygen as the main elements with 63.8% and 10.44%, respectively, in their compositions, which relate with the typical composition of cellulose [23]. This confirmed that the cellulose compartments for those NC were different as a result of the different hydrolysis treatment [24], as well as sulfur element with 22.25% that may deposit as a result of the acidic hydrolysis treatment, which indicates the applicable extraction of NC from palm tree fronds, for nitrogen, the weight was 3.5% may appear as result for fertilizers of the date palm in Iraq as a result of the harsh conditions that the country went through as wars, high salinity and pest invasion.

**Figure 5:**
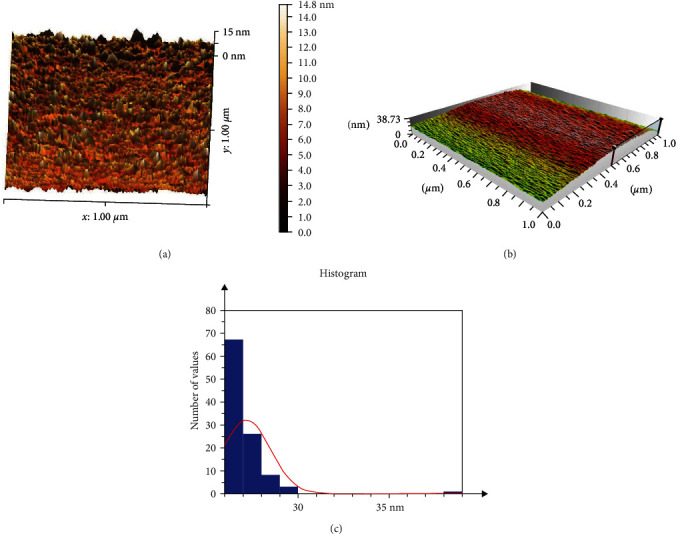
(a) 3D image of AFM for biosynthesized nanocellulose, scale bar: 1.00 *µ*m; (b) surface microgeometry and roughness parameters represented that roughness average is 7.20 nm with the root–mean-square (RMS) roughness value of 21.56 nm; (c) size distribution from histogram image of nanocellulsoe.

The 3D topographic image of AFM with taping mode illustrated a highly uniform distribution of particles and suggests that the size of the NC is ∼15 nm Figure 5(a). The statistical roughness analysis shows that the obtained roughness average is 7.20 nm with the root–mean-square (RMS) roughness value of 21.56 Figures 5(b) and 5(c), which corresponded relatively with the micrographs of SEM. The AFM results show that the roughness and the well-dispersed surface of NC; a previous study by Saud et al. [22] has reported a relatively similar finding. The RMS roughness is one of the characteristics of membrane surface topographies, and it was applied to study the surface roughness of the biosynthesized NC.

The literature by Kassab et al. [25] with 44.05 nm of RMS. AFM was used by Mohaiyiddin et al. [24] to analyze the morphology and topography of NC isolated from sugarcane debris. The extracted NC had a needlelike character with a uniform diameter and irregular length.

A study by Kassab et al. [25] presented that alkali with bleaching treatments leading to defibrillation with distinct microfibers revealing a flat surface by AFM due to noncellulosic compounds removal, different findings were used AFM examination to demonstrate that many features such as temperature, time, extraction process, pH value, and cellulose source influence the NC size with morphology.

## 4. Conclusion

The findings of the present study demonstrate the feasibility of utilizing acid hydrolysis to extract NC from date palm biowaste. The resulting nanoparticles exhibited a rod-like morphology, and their desirable nanosized ranging between 31 and 74 nm with a high content of *α*-cellulose was advantageous for nanocomposite processing. Consequently, the isolation of NC from date palm leaves offers a promising alternative for the development of NC products in the future.

## Figures and Tables

**Table 1:** The percent of chemical compositions for the biosynthesized nanocellulose from biowaste of date palm leaves.

Product	*α*-Cellulose (%)	Hemicellulose (%)	Lignin (%)
Nanocellulose	47.90	26.78	24.67

**Table 2:** Illustrated the weight precent for the elemental composition of the cellulosic nanoparticles extracted from palm tree fronds after acid hydrolysis treatment.

Element	wt.%	Pk/Bg	Lconf	Hconf
Nitrogen (N)	3.50	45.74	3.12	3.88
Oxygen (O)	63.80	201.20	62.47	65.14
Carbon (C)	10.44	49.60	9.89	10.99
Sulfur (S)	22.25	59.20	21.73	22.77
	100.00	—	—	—

## Data Availability

All data are available in the manuscript.
